# Rabbit Hemorrhagic Disease Virus in Mexico in 2020–2021: Risk Areas and Climatic Distribution

**DOI:** 10.3390/v16081344

**Published:** 2024-08-22

**Authors:** Consuelo Lorenzo, Jesús A. Fernández, Nathalie S. Hernández-Quiroz, Alberto Lafón Terrazas, Gloria Tapia-Ramírez

**Affiliations:** 1 El Colegio de la Frontera Sur, Unidad San Cristóbal, San Cristóbal de Las Casas C.P. 29290, Chiapas, Mexico; tapiaramglo@gmail.com; 2 Facultad de Zootecnia y Ecología, Universidad Autónoma de Chihuahua, Periférico Francisco R. Almada Km. 1, Chihuahua C.P. 31453, Chihuahua, Mexico; afernandezf@uach.mx (J.A.F.); nhernandez@uach.mx (N.S.H.-Q.); 3 Protección de la Fauna Mexicana, Calle 16, Número 2604, Colonia Pacífico, Chihuahua C.P. 31030, Chihuahua, Mexico; gruscan@yahoo.com.mx

**Keywords:** conservation, *Lepus*, RHDV2, *Sylvilagus*, virus

## Abstract

Mexico is home to 14 species of lagomorphs, 6 of which are endemic. Studies on diseases affecting native lagomorphs are scarce, and in most cases, the impact on their populations remains largely unknown. Rabbit hemorrhagic disease virus (RHDV), especially the RHDV2 variant, causes a serious and extremely contagious disease, resulting in high mortality rates and major declines in wild lagomorph populations. The objectives of this study were to identify disease hotspots and critical biodiversity regions in Mexico through the combined use of disease information and lagomorph distribution maps and to determine the areas of greatest concern. In total, 19 states of Mexico recorded RHDV2 from April 2020 to August 2021, and 12 of them reported the wild species *Sylvilagus audubonii*, *Lepus californicus*, and unidentified Leporidae species. The distribution of RHDV2 in Mexico can be closely predicted from climatic variables. RHDV2 hotspots are located in the central-southern area of the Mexican Highlands and the Trans-Mexican Volcanic Belt, where the virus affects multiple species. This knowledge is essential for proposing specific actions to manage and preserve lagomorph populations at risk and address these issues as soon as possible.

## 1. Introduction

Worldwide, there is a high taxonomic diversity (93 species) of pikas, rabbits, and hares (order Lagomorpha) [1]. Part of this diversity is represented in the Americas, home to 5 of the 12 lagomorph genera existing in the world: *Ochotona* (pikas), *Lepus* (hares), and *Brachylagus*, *Romerolagus*, and *Sylvilagus* (rabbits). Likewise, many other unique endemic lagomorph species are found in other countries, regions, and ecosystems. Lagomorphs undoubtedly represent an important component of global biodiversity.

Mexico ranks third in lagomorph diversity worldwide, with 14 species, 6 of which are endemic. Unfortunately, several of its wild populations are declining at an accelerated rate due to the adverse impact of human activities on the natural environment of this group of species. Activities with the greatest impact include the introduction of exotic species (dogs and cats as predators and goats as competitors for food and space), hunting, trade in their meat and skin, and activities that modify or destroy their habitat, such as livestock ranching, agriculture, induced fires, and urban development. Climate change is an additional risk factor that threatens the survival of lagomorphs [1]. For this reason, numerous species of wild rabbits and hares in Mexico are listed in an extinction risk category on the International Union for Conservation of Nature (IUCN) Red List of Threatened Species [2], mainly those distributed in restricted geographic areas of less than 300 km^2^ or on islands [3].

Additional aspects to consider for these groups of species is the lack of information of their biology and natural history. Studies addressing the diseases that affect them are scarce and focus on the European rabbit (*Oryctolagus cuniculus*), given its commercial value. However, the effect of diseases caused by pathogens such as insects and viruses on populations of wild lagomorph species remains unknown. Some of these microorganisms are highly infectious and easily dispersed (by other animals and humans). Such is the case of rabbit hemorrhagic disease virus (RHDV), which emerged as the RHDV2 variant in the Americas in 2020 [4]. RHDV2 causes a highly contagious hemorrhagic reaction and sudden death in lagomorphs. It was first detected in domestic rabbits (*O. cuniculus*) in France in 2010 and has since spread to other European and non-European countries, where it has also caused heavy mortality in wild rabbits. RHDV2 outbreaks have been recorded in the Americas since 2016 in Quebec, Canada, then in March 2018 in British Columbia, Canada, and later in the United States (Washington, Ohio, and New York), affecting feral domestic rabbits, *Oryctolagus cuniculus*. The 2020 RHDV2 outbreak was particularly significant because it concerned the infection and subsequent spread of the disease in native lagomorphs [5,6].

Rabbit hemorrhagic disease (RHD) is a serious and extremely contagious disease that causes high mortality rates (between 40% and 100% for RHDV and between 5% and 80–90% for RHDV2), leading to major declines in wild populations [7,8,9]. In lagomorph populations, this disease has resulted in serious ecological imbalances with catastrophic consequences on biodiversity, since lagomorphs play key roles in the food chain as herbivores that regulate carnivore population cycles and as seed carriers that contribute to seed dispersal. Additionally, several lagomorph species are used as a source of food in rural communities [10], so their economic importance is also reduced. In addition, the decline of lagomorph populations can increase carnivore predation on domestic livestock [11].

In March 2020, RHDV2 was recorded in the state of New Mexico, USA, and within three months, it was also detected in multiple counties in the southwestern US states of Arizona, California, Colorado, Nevada, Texas, and Utah [12]. Subsequently, it was also reported in northern Mexico, in several municipalities of northern Chihuahua, northeast and south-central Durango, central Sonora, northern Baja California Sur, and northwest Baja California [4]; multiple native species of *Sylvilagus* and *Lepus* species and pygmy rabbits (*Brachylagus idahoensis*) have been documented as affected [12,13].

Knowing the occurrence of RHDV2 cases in Mexico and their evolution, and determining the wild species involved and the areas at highest risk of RHDV2, is vitally important to propose specific actions to manage and preserve wild lagomorph populations at risk. The objectives of this study were to gather the records of RHDV2 cases in Mexico from April 2020 to August 2021, and to identify climatic factors determining the spread of RHDV2 in relation to lagomorph biodiversity hotspots and the number of rabbit and hare species involved, highlighting those listed as endangered or in a risk category.

## 2. Materials and Methods

### 2.1. Lagomorph Species Compilation

The current distribution and endemism of lagomorphs in Mexico according to [1] were reviewed considering recent taxonomic changes [14,15,16] and their extinction risk category according to the IUCN Red List of Threatened Species [2].

### 2.2. Collation of RHDV2 Case Data

After reviewing reports available for 2020 and 2021 (since more recent data are not available) at the World Organisation for Animal Health database (https://www.oie.int/wahis_2/public/wahid.php/Reviewreport/Review?reportid=35948, accessed on 7 March 2024) [8], we identified the locations, geographic coordinates, and dates where RHDV2 cases have occurred, as well as the wild and domestic lagomorph species affected, the numbers of animals involved, and the control measures applied in each case.

Samples were collected from dead animals and analyzed in the Level-3 Biosafety Laboratory of the National Service of Health, Safety, and Agrifood Quality (Servicio Nacional de Sanidad, Inocuidad y Calidad Agroalimentaria; SENASICA, in Spanish) of the Mexican government. These samples tested positive for rabbit hemorrhagic disease using the hemagglutination inhibition assay for antibody detection (Ab HI) and reverse transcription polymerase chain reaction (RT-PCR).

### 2.3. Determination of Lagomorph Species Richness Hotspots

The potential distribution polygons for lagomorph species in Mexico available from the Map of Life [17] website (https://mol.org/species, accessed on 7 March 2024) were used, based on the proposal of [18], as well as those proposed by the IUCN (https://www.iucnredlist.org/, accessed on 7 March 2024). The distribution polygons were fitted to the Mexican territory and superimposed using Geographic Information Systems (GIS) to visualize the degree of coincidence of species. Polygons with high species overlap were considered hotspots.

### 2.4. Determination of the Climatic Probability of Occurrence of Lagomorphs Affected by RHDV2

The potential RHDV2 climatic distribution models for affected domestic and wild lagomorphs was constructed using the available information on cases involving affected wild and domestic animals from the World Organisation for Animal Health database. Two potential distribution models were run, one for domestic animals and the other for wild animals. The geographic coordinates (LATLONG) were extracted for each record, and the information on climate data layers was downloaded from the WorldClim platform (https://www.worldclim.org, accessed on 7 March 2024). Of the 19 layers of climate variables available, we used information at a spatial resolution of 30 s (~1 km^2^). For each record (set of coordinates), the estimated value of each climatic variable was extracted. The information was sorted by the type of affected animal (domestic or wild), and a Spearman correlation analysis was run on each data set to exclude redundant (correlated) variables in the model; the threshold value used was 0.75. From the correlated variables, we selected the one most relevant for the virus or that explains its behavior.

The model was generated with the MaxENT 3.x software using the bootstrap process with 100 replicates. In total, 75% percent of the data were used to generate the model and the remaining 25% for validation. This method produces files in raster format that show the probability of occurrence of the virus; in this study, for a better visualization of the information, probability values were sorted into quartiles every 0.25, and values lower than 0.1 were considered as a zero probability.

## 3. Results

### 3.1. Lagomorph Species List

In total, 14 lagomorph species have been recorded in Mexico: 8 species of *Sylvilagus*, 5 of *Lepus*, and 1 of *Romerolagus*. Of these, 6 species are endemic: *R. diazi*, *S. cunicularius*, *S. graysoni*, *S. insonus*, *L*. *flavigularis*, and *L. altamirae* (Table 1). In addition, according to the IUCN Red List of Threatened Species [2], 7 species are listed as Least Concern, 2 Vulnerable, 3 Endangered, 1 Data Deficient, and 1 Undetermined (not listed yet) (Table 1).

### 3.2. RHDV2 Case Data

**Figure 1:**
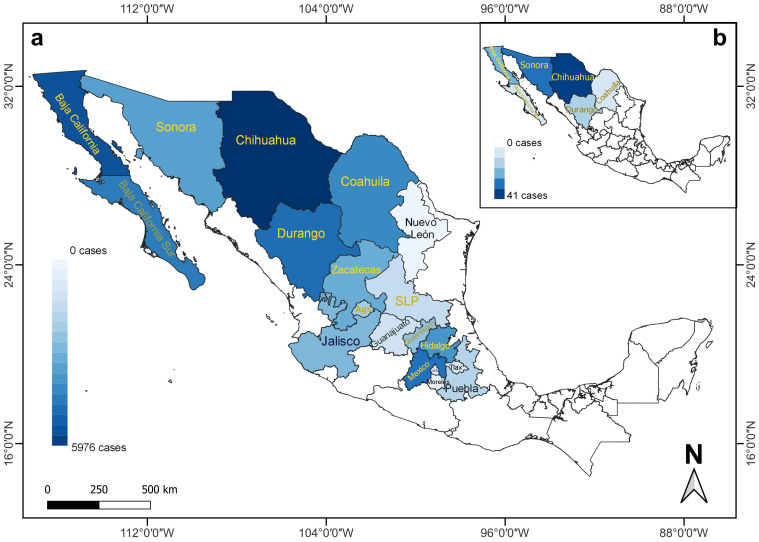
Number of RHDV2 cases recorded for lagomorph species (*Oryctolagus cuniculus*, wild and unidentified species) in different states of Mexico between (**a**) April 2020 and August 2021, and (**b**) March 2020 and April 2020 [8], illustrating the rapid expansion of the disease toward southern Mexico. The different shades of blue vary depending on the number of cases. States with names in yellow (*n* = 12 in (**a**); *n* = 6 in (**b**)) indicate the presence of RHDV2 cases in wild species. Ags (Aguascalientes), CdMx (Ciudad de Mexico), SLP (San Luis Potosi), and Tlax (Tlaxcala).

In Mexico, 1070 RHDV2 cases were recorded in the states located in the north, the Baja California Peninsula, and the center of the country between April 2020 and August 2021. RHDV2 cases occurred in 19 states (Figure 1 and Table 2), and 12 affected wild species (Figure 1). No available data on RHDV2 cases were reported after August 2021.

**Figure 2:**
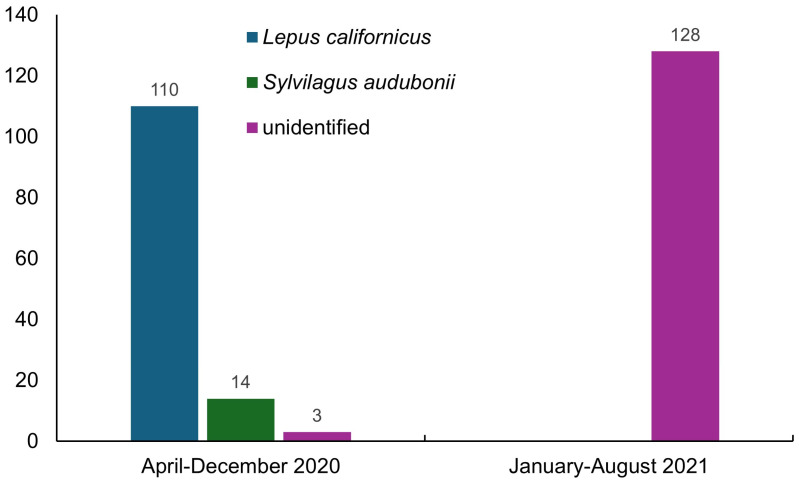
Number of RHDV2 cases diagnosed in wild and unidentified species in Mexico between April 2020 and August 2021 [8].

**Figure 3:**
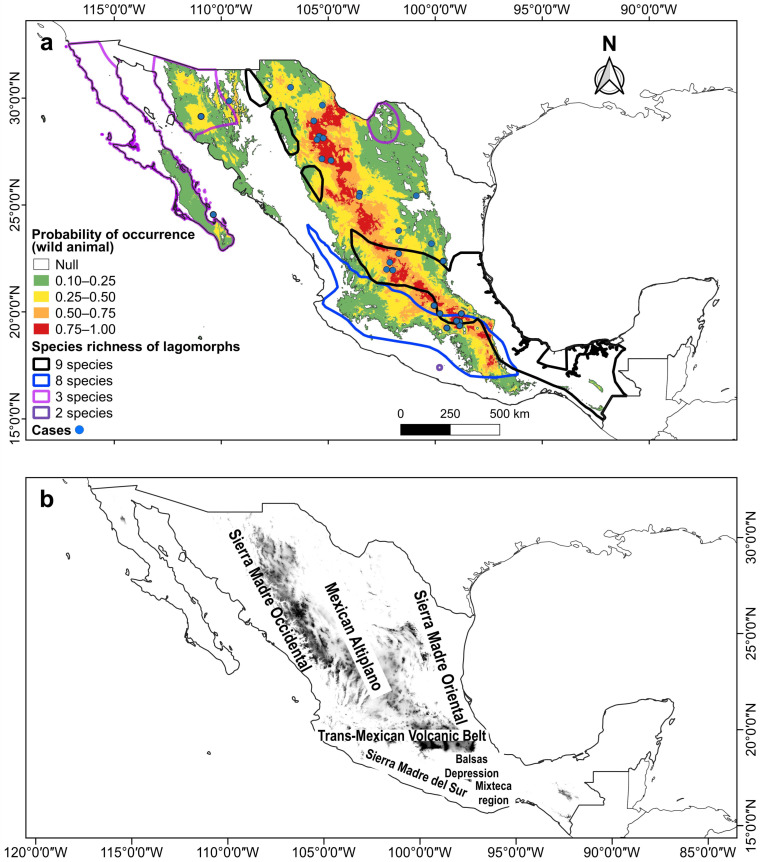
(**a**) Combined maps of the probability of occurrence of the potential climatic distribution of RHDV2, species richness areas, and RHDV2 cases (records in blue dots) in wild lagomorph species in Mexico. (**b**) Relief of Mexico with the names of the main mountain ranges and plateaus.

**Figure 4:**
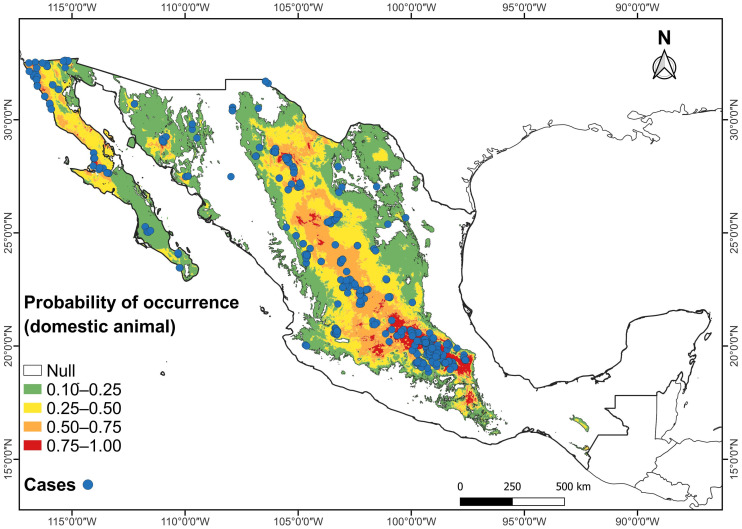
Combined maps of probability of occurrence of the potential climatic distribution of RHDV2 and RHDV2 cases (records in blue dots) in the domestic rabbit *Oryctolagus cuniculus* in Mexico.

Considering all records, we found 27,062 cases of affected animals (Table 2), 255 corresponding to wild species (Figure 2 and Figure 3) and 26,807 to domestic *O. cuniculus* (Figure 4).

Two species of wild leporids affected by RHDV2 have been recorded in Mexico, as well as unidentified leporids, reported as follows by state: 14 *Sylvilagus audubonii* (12 in Chihuahua, 2 in San Luis Potosi), 110 *Lepus californicus* (82 in Chihuahua, 7 in Sonora, 6 in Coahuila, 5 in Zacatecas, 4 in Aguascalientes, 3 in Baja California Sur, 2 in San Luis Potosi, and 1 in Durango), and 131 unidentified leporids (91 in Mexico, 30 in Hidalgo, 3 in Baja California, 2 in each of Baja California Sur, Queretaro, and Sonora, and 1 in San Luis Potosi) (Figure 1a).

### 3.3. Lagomorph Species Richness Hotspots

Two areas where high RHDV2 incidence overlapped with high lagomorph species richness were identified. One includes up to nine species (*Lepus altamirae*, *L. californicus*, *L. callotis*, *L flavigularis*, *Romerolagus diazi*, *Sylvilagus audubonii*, *S. cunicularius*, *S. floridanus*, and *S. gabbi*) (Figure 3a) in fragmented areas of the Gulf coastal plain, the Trans-Mexican Volcanic Belt, and the southern-central part of the Sierra Madre Oriental (Figure 3b). The other area harbors up to eight species (*L. alleni*, *L. californicus*, *L. callotis*, *S. audubonii*, *S. cunicularius*, *S. floridanus*, *S. gabbi*, and *Romerolagus diazi*) (Figure 3a) in the Sierra Madre del Sur and the Sierra Madre Occidental (Figure 3b). Other overlap areas where three species coexist are located in the north of the country: the Baja California Peninsula (*S. audubonii*, *S. bachmani*, and *L. californicus*), northern and central Sonora (*L. alleni*, *L. californicus*, and *S. audubonii*), and northern Coahuila (*L. californicus*, *S. audubonii*, and *S. robustus*), and there is another where the distribution of two species overlaps in the Mixteca region in northern Oaxaca (*S. cunicularius* and *S. insonus*) (Figure 3).

### 3.4. Climatic Probability of Occurrence of Lagomorphs Affected by RHDV2

The climatic variables that best explain the probability of occurrence of domestic rabbits affected by RHDV2 in Mexico are BIO 7 (annual temperature range), BIO 8 (mean temperature of the wettest quarter), BIO 9 (mean temperature of the driest quarter), BIO 11 (mean temperature of the coldest quarter), BIO 12 (annual precipitation), BIO 15 (precipitation seasonality), BIO 18 (precipitation of the warmest quarter), and BIO19 (precipitation of the coldest quarter). In the case of affected wild rabbits and hares, these variables are BIO 2 (mean diurnal range), BIO 7 (annual temperature range), BIO 9 (mean temperature of driest quarter), BIO 10 (mean temperature of the warmest quarter), BIO 11 (mean temperature of the coldest quarter), BIO 12 (annual precipitation), BIO 14 (precipitation of the driest month), BIO 15 (precipitation seasonality), and BIO 19 (precipitation of the coldest quarter).

The highest probability of occurrence (0.75–1.0) within the potential climatic distribution of RHDV2 in Mexico for both *O. cuniculus* and wild lagomorph species occurs in the plateau known as the Altiplanicie Mexicana (Mexican Altiplano). This plateau stretches from the state of Chihuahua in the north to the State of Mexico and Puebla in central Mexico; in this area, it meets the Trans-Mexican Volcanic Belt, which extends from east to west between the parallels 19° and 20° N (Figure 3 and Figure 4). The Mexican Altiplano slopes towards the coastal plains of the Gulf of Mexico; its climate varies from warm and dry at the lower altitudes in the north to rainy with cold winters in the south. The Mixteca region, in northern Oaxaca, also shows a high probability of occurrence (Figure 3 and Figure 4).

## 4. Discussion

### 4.1. RHDV2 Case Data for Lagomorph Species

In 2020, only 6 states of northern Mexico were known to be affected by RHDV2 (Baja California, Baja California Sur, Chihuahua, Coahuila, Durango, and Sonora) [4]; however, this disease was recorded in 19 states in 2021, indicating its rapid spread to tropical latitudes below 24° N. These data also document the lack of control measures to prevent its spread and dissemination to different social levels through contagion. In addition, the number of cases of the disease in lagomorphs increased considerably, from 4053 (21 in wild species) in April 2020 [4] to 27,062 (255 in wild species) between this date and August 2021. The annual number of RHDV2 cases involving the domestic species *O. cuniculus* decreased from 19,361 cases in 2020 to 7446 in 2021. In contrast, there was a slight increase in the number of cases in wild species (*L. californicus*, *S. audubonii*, and unidentified species), from 117 in 2020 to 128 in 2021; however, it is worth noting that in the latter year, no details of the species affected were published by the World Organisation for Animal Health. The cases reported in wild species observed in the field were sporadic and incidental, without any systematic and continuous monitoring of the behavior of the disease in wild populations over time. The affected species are currently unknown, having only been recorded as unidentified species, which stresses the current lack of awareness in Mexico about this disease and the relevance of its potential impact on endemic and endangered species.

The records of RHDV2 cases show a similar pattern of distribution for domestic rabbits and wild lagomorph species. This similarity is likely because the data used for detecting the patterns came from areas adjacent to the locations of cases involving domestic rabbits, which are probably closely monitored. Therefore, the number of cases in wild animals is probably under-represented throughout the country due to the technical and human limitations preventing the inclusion of most of its distribution area. Additionally, there is scarce information, if any, about this topic at the country level. For the above, continuous long-term monitoring is needed, involving sufficient technical and human resources to allow the identification of potential changes in the distribution of lagomorph species and the RHDV2 virus.

### 4.2. Lagomorph Species Richness Hotspots

In Mexico, the RHDV2 vulnerability hotspots, which are the areas of greatest concern in the country, are located in the southern-central part of the Mexican Altiplano and the Trans-Mexican Volcanic Belt; in these areas, the virus could affect up to nine lagomorph species. The species that may potentially be affected and for which there are no records of the disease include the zacatuche rabbit, *R. diazi*, in the Trans-Mexican Volcanic Belt; *L. callotis* (almost endemic to Mexico), with a fragmented distribution in the Sierra Madre Occidental and central Mexico; *S. cunicularius* (endemic to Mexico), recorded in the Balsas Depression and southern areas of the Sierra Madre Oriental; and *S. floridanus*, a species inhabiting central and southern Mexico.

It is important to recognize that because of the easy dispersal of RHDV2, a highly infectious virus resistant to environmental changes, wild lagomorph species or populations that live on islands may also be affected by the disease. This is the case of hares endemic to the Baja California Sur islands [19]: *L. californicus insularis* on Espiritu Santo Island, *L. c. sheldoni* on Carmen Island, *L. c. magdalenae* on Magdalena and Margarita Islands, *L. californicus* on Cerralvo Island [20], and *L. alleni tiburonensis* on Isla Tiburón, Sonora; and the rabbits *S. bachmani mansuetus* on Isla San José, Baja California Sur, *S. b. cerrosensis* on Isla Cedros in Baja California [19], and *S. graysoni* in Islas Marías, Nayarit. Other species of great importance in conservation are the Omiltemi rabbit, *S. insonus*, in the Sierra Madre del Sur; the Altamira hare, *L. altamirae*, in the Gulf coastal plain in southern Tamaulipas; and the Tehuantepec hare, *L. flavigularis*, around the lagoon area of the southern Isthmus of Tehuantepec, Oaxaca. These species are endemic, are listed in a risk category, and require continuous monitoring to evaluate whether they are affected by RHDV2, although according to our modelling, the likely occurrence of RHDV2 is extremely low.

### 4.3. Determination of the Climatic Probability of Occurrence of Lagomorphs Affected by RHDV2

The highest probability of occurrence of domestic rabbits, *O. cuniculus*, affected by RHDV2 in Mexico coincides with the records of the presence of the disease on farms, backyard farms, veterinary clinics, parks, laboratories, and modernized farms, and in pets. Due to the easy spread of the disease, we found it interesting to include the known records involving the domestic rabbit, as they likely follow the same distribution pattern as those of wild species.

It has been observed that arid and semiarid regions in the western US are the worst areas affected by RHDV2, in contrast with humid regions in the eastern US [13,21]. The present study revealed a coincidence in the distribution of RHDV2 cases in domestic and wild lagomorphs in arid and semiarid areas of the Mexican Altiplano, from northern Mexico to central areas of the country. This region has the highest number of cases (307 in farms for *O. cuniculus* and 9 for wild species), and the Baja California Peninsula shows a similar pattern of high RHDV2 incidence. These areas show an overlap of the distribution of several wild Mexican lagomorph species, some of which are endemic to the country.

The epidemiological follow-up Report 36 issued by the World Organisation for Animal Health (dated 12 January 2022) states that type-2 rabbit hemorrhagic disease (RHD-T2) was declared endemic in 2021, and a vaccine was approved by the Mexican Government and made available at no charge to immunize domestic rabbits against the disease. Note that 187 cases were reported in 2020 and 230 in 2021 across 19 states, and that approximately 400,170 doses of vaccine have been supplied in this period. Currently, there are no vaccines or management plans to contain the disease in wild species.

The present study documented that the highest probability of occurrence of the potential climatic distribution of RHDV2 in Mexico is similar in domestic rabbits and wild lagomorph species.

According to the World Organisation for Animal Health monitoring reports in Mexico, the control measure applied for wild lagomorph species has been surveillance within and outside the containment or protection zone, the official elimination of infection routes (carcasses), byproducts, and wastes of animal origin, and sanitary culls [8].

## 5. Conclusions

Having more up-to-date data on RHDV2 cases is extremely important to accurately assess the current risk, improve predictive models, and implement effective control measures to protect vulnerable rabbit and hare populations. Therefore, efforts should be made to obtain field records of wild species with precise location data and a set of photographs useful for lagomorph species identification. This would enable the degree of impact on species with a restricted distribution, listed in a risk category, or endemic. Additionally, the movement of people onto islands should be restricted since RHDV2 contagion can be facilitated through footwear and clothing. For example, on María Madre Island in the Marías Islands Archipelago (where *S. graysoni* occurs), people live there permanently, and it has received tourism since it was declared a Biosphere Reserve in March 2019, ceasing to be the Islas Marías Federal Penal Colony [22]. For collaboration with international partners, it is essential to have accurate records of outbreak dates. Also, social networks should be used to report sightings of diseased or dead wild rabbits and hares, promoting the participation of farmers, hunters, gatherers, and the general public. This information may be useful to identify the locations where affected animals are detected, implement biosecurity measures to contain RHDV2 in wild lagomorph populations, and carry out joint management actions to prevent the spread of this disease.

## Figures and Tables

**Table 1:** Lagomorph species occurring in Mexico, distribution, and conservation status according to the IUCN Red List of Threatened Species [2].

Species	Country	County/State/Department	Conservation Status
*Romerolagus diazi* *	Mexico	Mexico City, Puebla	Endangered
***Sylvilagus audubonii***	United States, Mexico	Western Great Plains, from the southern border of Canada to Mexico City. To the west, from central California, Nevada, Utah, Baja California Peninsula, coastal Sonora, and Sonoran and Chihuahuan Deserts	Least Concern
***Sylvilagus bachmani***	United States, Mexico	Pacific coast from the Columbia River to the tip of the Baja California Peninsula, San Jose Island	Least Concern
*Sylvilagus cunicularius* *	Mexico	Sinaloa, Nayarit, Jalisco, Colima, Michoacan, Western Guerrero, Oaxaca, State of Mexico, Mexico City, Morelos, Hidalgo, Tlaxcala, Puebla, Veracruz	Least Concern
***Sylvilagus floridanus***	Southern Canada, eastern United States, Mexico, Guatemala, El Salvador, Honduras, Nicaragua, northern Costa Rica, northern Colombia, northern Venezuela	In Mexico: Aguascalientes, Campeche, Chiapas, Chihuahua, Coahuila, Durango, Guanajuato, Hidalgo, Jalisco, Mexico City, State of Mexico, Michoacan, Morelos, Nayarit, Nuevo Leon, Oaxaca, Puebla, Queretaro, Quintana Roo, San Luis Potosi, Sinaloa, Sonora, Tabasco, Tamaulipas, Tlaxcala, Veracruz, Yucatan, Zacatecas	Least Concern
*Sylvilagus gabbi*	Southeastern Mexico, Colombia	Southeastern Mexico (Veracruz, Tabasco, Chiapas), Guatemala, Southern Belice, Honduras, Nicaragua, Costa Rica, Panama, northwestern Colombia	Least Concern
*Sylvilagus graysoni* *	Mexico	Tres Marias Islands, Nayarit	Endangered
*Sylvilagus insonus* *	Mexico	Southern and central Guerrero, Sierra Madre del Sur, Omiltemi State Park	Data Deficient
*Sylvilagus robustus*	United States, Mexico	Southern New Mexico, southwestern Texas, northern Coahuila	Vulnerable
***Lepus alleni***	United States, Mexico	Arizona, Sonora, western Chihuahua, Sinaloa, northern Nayarit, Tiburon Island	Least Concern
*Lepus altamirae* *	Mexico	Central and southern Tamaulipas, Gulf of Mexico coast	Undetermined
***Lepus californicus***	United States, Mexico	Two-thirds of western and central United States, 15 states of Mexico, Espiritu Santo Island, Cerralvo Island	Least Concern
*Lepus callotis*	United States, Mexico	Southwestern New Mexico, western Chihuahua, Durango, southern Zacatecas, southwestern Nayarit, Jalisco, northern Colima, Michoacan, southern San Luis Potosi, Guanajuato, central and southern Queretaro, southern Hidalgo, State of Mexico, Mexico City, Morelos, Tlaxcala, Puebla, northern Guerrero, northwestern Oaxaca	Vulnerable
*Lepus flavigularis* *	Mexico	Southeastern Oaxaca	Endangered

* = species endemic to Mexico. In bold = species affected by RHDV2 in North America.

**Table 2:** Number of RHDV2 cases recorded for lagomorph species (*Oryctolagus cuniculus*, wild and unidentified species) in different states of Mexico between April 2020 and August 2021. See also Figure 1.

State	Number of Cases
Aguascalientes	358
Baja California	3104
Baja California Sur	2339
Chihuahua	5976
Coahuila	2257
Durango	2423
Guanajuato	147
Hidalgo	2221
Jalisco	1087
Mexico City	122
Nuevo Leon	2
Puebla	241
Queretaro	362
San Luis Potosi	194
Sonora	1980
State of Mexico	2518
Tlaxcala	122
Zacatecas	1602
Total	27,062

## Data Availability

The original contributions presented in the study are included in the article, further inquiries can be directed to the corresponding author.

## References

[1] Smith A.T., Johnston C.H., Alves P.C., Häcklander K. (2018). Lagomorphs: Pikas, Rabbits and Hares of the World.

[2] IUCN (International Union for Conservation of Nature) The IUCN Red List of Threatened Species 2023-1. https://www.iucnredlist.org/.

[3] Lorenzo C., Rioja-Paradela T.M., Carrillo-Reyes A. (2015). State of knowledge and conservation of endangered and critically endangered lagomorphs worldwide. Therya.

[4] Lorenzo C., Lafón-Terrazas A., Fernández J.A., Cervantes F.A., Martínez-Meyer E. (2020). La enfermedad hemorrágica viral del conejo impacta a México y amenaza al resto de Latinoamérica. Therya.

[5] National Wildlife Health Center Rabbit Hemorrhagic Disease Virus 2 Confirmed in Wild Rabbits in the United States. https://www.usgs.gov/centers/nwhc/science/wildlife-health-bulletins?qt-science_center_objects=0#qt-science_center_objects.

[6] Miller R.E., Calle P.P., Lamberski N. (2022). USDA Notices or RHDV2 Book Chapter 25 in Fowler’s Zoo and Wild Animal Medicine Current Therapy.

[7] Ahmad S.T., El-Samadony H.A., Mahgoub K.M. (2011). Immunological and Virological Studies on Rabbit Hemorrhagic Disease Virus. Glob. Vet..

[8] The Center For Food Security & Public Health, Institute For International Cooperation in Animal Biologics, Iowa State University, World Organization For Animal Health (OIE), United States Department of Agriculture (USDA) (2016). Rabbit Hemorrhagic Disease. http://www.cfsph.iastate.edu/Factsheets/pdfs/rabbit_hemorrhagic_disease.pdf.

[9] House Rabbit Society Rabbit Hemorrhagic Disease Virus (RHDV). https://rabbit.org/rhdv/.

[10] Lorenzo C., Romero A. (2012). Importancia biológica de los lagomorfos. Therya.

[11] Grajales T., González R.A. (2014). Determinación de la dieta estacional del coyote (*Canis latrans*) en la región norte de la Reserva de la Biosfera Mapimí, México. Rev. Mex. Biodivers..

[12] Impacted Species-RHDV2.org RHD Awareness Team. https://rhdv2.org/impacted-species/.

[13] Cima G. Rabbit Hemorrhagic Disease Virus Serotype 2 Spreading among Wild Rabbits, Hares. https://www.avma.org/javma-news/2020-07-15/virus-killing-rabbits-western-us.

[14] Álvarez-Castañeda S.T., Lorenzo C. (2016). Genetic evidence supports *Sylvilagus mansuetus* (Lagomorpha: Leporidae) as a subspecies of *S. bachmani*. Zootaxa.

[15] Álvarez-Castañeda S.T., Lorenzo C. (2017). Phylogeography and phylogeny of *Lepus californicus* (Lagomorpha:Leporidae) from Baja California Peninsula and adjacent islands. Biol. J. Linn. Soc..

[16] Vargas K., Brown D., Wisely E., Culver M. (2019). Reinstatement of the Tamaulipas white-sided jackrabbit, *Lepus altamirae*, based on DNA sequence data. Rev. Mex. Biodivers..

[17] Map of Life (2021). Mammal Range Maps Digitized from the Illustrated Checklist of the Mammals of the World (Burgin et al., 2020). https://mol.org/species.

[18] Burgin C.J., Wilson D.E., Mittermeier R.A., Rylands A.B., Lacher T.E., Sechrest W. (2020). Illustrated Checklist of the Mammals of the World.

[19] Lorenzo C., Rioja-Paradela T., Carrillo-Reyes A., de la Paz-Cuevas M. (2018). Conejos y Liebres Insulares de México. Insular Rabbits and Hares of Mexico.

[20] Lorenzo C., Álvarez-Castañeda S.T., Cortés-Calva P., de la Paz M., Bolaños J.E. (2010). Status of an invading mainland jackrabbit on Cerralvo Island. Gulf of California. West. N. Am. Nat..

[21] Ringenberg J.M., Weir K., Linder T., Lenoch J. (2024). Detections of Rabbit Hemorrhagic Disease Virus 2 (RHDV2) Following the 2020 Outbreak in Wild Lagomorphs across the Western United States. Viruses.

[22] Diario Oficial de la Federación (DOF) 08/03/2019. DECRETO por el que se Desincorporan del Sistema Federal Penitenciario los Centros Federales de Readaptación Social que se Indican, Ubicados en el Complejo Penitenciario Islas Marías. https://www.dof.gob.mx/nota_detalle.php?codigo=5552278&fecha=08/03/2019#gsc.tab=0.

